# Dispensation of attention deficit hyperactivity disorder (ADHD) medications in patients receiving opioid agonist therapy; a national prospective cohort study in Norway from 2015 to 2017

**DOI:** 10.1186/s12888-020-02526-y

**Published:** 2020-03-12

**Authors:** Jørn Henrik Vold, Christer Aas, Svetlana Skurtveit, Ingvild Odsbu, Fatemeh Chalabianloo, Anne Halmøy, Kjell Arne Johansson, Lars Thore Fadnes

**Affiliations:** 1 Department of Addiction Medicine, Haukeland University Hospital, Bergen, Norway; 2 Department of Global Public Health and Primary Care, University of Bergen, Bergen, Norway; 3 Department of Mental Disorders, Norwegian Institute of Public Health, Oslo, Norway; 4 Norwegian Centre for Addiction Research, University of Oslo, Oslo, Norway; 5 Department of Medicine, Karolinska Institutet, Stockholm, Sweden; 6 Division of Psychiatry, Haukeland University Hospital, Bergen, Norway; 7 Department of Clinical Medicine, University of Bergen, Bergen, Norway

**Keywords:** Opioid substitution treatment, Centrally acting stimulants, Hyperkinetic disorder, Attention deficit hyperactivity disorder, Substance-related disorders, Dispensed drugs, Register data

## Abstract

**Background:**

It is estimated that up to a third of patients on opioid agonist therapy (OAT) have attention deficit hyperactivity disorder (ADHD). Treatment by ADHD medication, including a centrally acting stimulant (CAS) or atomoxetine is one of the essential approaches. This study evaluates the use of dispensed ADHD medications in the Norwegian OAT population in the period from 2015 to 2017. Types and doses of ADHD medications, co-dispensations of other potentially addictive drugs like benzodiazepines, z-hypnotics, gabapentinoids, and non-OAT opioids, as well as direct-acting antivirals (DAA) against hepatitis C infection, are investigated.

**Methods:**

Information about all dispensed ADHD medication, OAT opioids, and the defined potentially addictive drugs were recorded from the Norwegian Prescription Database. Dispensation rates, the types, and the doses of dispensed ADHD medications were estimated by summarizing the number of dispensations, and the dispensed doses. Logistic regression analyses were employed to assess the associations between ADHD medication, and OAT opioid use, and dispensations of other potentially addictive drugs and DAAs against hepatitis C infection.

**Results:**

A total of 9235 OAT patients were included. The proportion of patients who were dispensed ADHD medication increased from 3.5 to 4.6% throughout the study period. The three most dispensed CAS were short- and intermediate-acting methylphenidate (55%), lisdexamphetamine (24%), and dexamphetamine (17%) in 2017. Buprenorphine, rather than methadone, as OAT opioid (adjusted odds ratio: 1.6, CI: 1.2–2.1) was associated with being dispensed ADHD medication. Among patients who received CAS and OAT opioids each calendar year, the dispensed doses of methylphenidate increased from 63 mg/day in 2015 to 76 mg/day in 2017 (*p* = 0.01). Sixty percent of patients receiving ADHD medications were also dispensed other addictive drugs concomitantly in 2017. Similar results were found in 2015 and 2016.

**Conclusion:**

Co-prescription of ADHD medications was low among patients on OAT in Norway, considering a high prevalence of ADHD in this patient group. On the other hand, concurrent dispensations of multiple addictive drugs were common in this population. Understanding the underlying reasons for such prescribing is essential, and research on how to optimize ADHD medication of patients with ADHD receiving OAT is needed.

## Background

The strong association between opioid addiction and attention deficit hyperactivity disorder (ADHD) is well known [1]. Studies indicate that up to a third of patients receiving opioid agonist therapy (OAT) meet the criteria for ADHD [1–5]. Both opioids used in OAT and centrally acting stimulants (CAS) may have properties associated with euphoria and addiction. Current treatment guidelines and previous reviews, therefore, recommend stable psychosocial surroundings and close follow-ups by health professionals in case of prescription of these high-potent drugs [6–10]. The use of other reinforcing, potentially addictive drugs such as benzodiazepines, z-hypnotics, non-OAT opioids, and gabapentinoids should be considered carefully to prevent adverse interactions and risk of new addictions [11, 12]. However, about 50% of patients receiving OAT and ADHD medication, including CAS and atomoxetine, concomitantly discontinue ADHD medication during the first 2 years after the start of the treatment [13]. Reasons for discontinuation include illicit drug use, side effects, and lack of psychosocial stability [13]. Long-term therapy of ADHD medication seems to have the highest chance of adequate adherence when combining psychosocial treatment with ADHD medication and OAT in the absence of other reinforcing, potentially addictive drugs [14, 15].

Little is known about the prevalence of co-existing ADHD and the utilization and the dose of prescribed ADHD medication among patients with ADHD on OAT. Additional use of other potentially addictive drugs makes ADHD assessment and treatment with ADHD medications more challenging. Psychosocial factors and medical conditions among these patients may also complicate diagnosis and the co-therapy with ADHD medication. Therefore, studies show substantial inter-country differences in co-existing ADHD prevalence (5–30%) [2, 16], and variations in utilization and the dose of prescribed ADHD medication [7, 9, 10, 17, 18]. There is evidence that CAS have an effect by suppressing ADHD symptoms among patients with drug use disorders and comorbid ADHD [18]. Some studies also point towards that the high-dosed CAS increases patients’ retention to treatment, and prevents discontinuation [19, 20]. However, individual assessment taking into consideration medical and psychosocial conditions will be of particular interest to ensure a proper prescription of CAS to patients on OAT with comorbid ADHD.

During the past years, the guidelines for ADHD worldwide recommend prescribing ADHD medication to patients on OAT with comorbid ADHD and those with other drug use disorders if they are abstinent from any illegal drugs [7, 9, 10, 17]. However, the evidence supporting this recommendation is weak. In Norway, prescribing ADHD medication has been recommending for patients with ADHD on the OAT program since 2014 [21]. A total of 7500 Norwegian patients are given OAT [22], and, in 2016, about 15% self-reported the use of illegal and legal CAS during the last 4 weeks. Although the proportion that was dispensed ADHD medication, on medical indication is uncertain after the guidelines were revised. To be able to improve the treatment of ADHD, it is essential to know more about the current prescription rates of ADHD medications and the prescription patterns of other potentially addictive drugs among patients on OAT who were dispensed an ADHD medication.

Thus, this observational study was aimed to define dispensation rates of attention deficit hyperactivity disorder (ADHD) medications and potentially addictive drugs among patients on opioid agonist therapy (OAT) in the period from 2015 to 2017 in Norway. The aims were to:

1. define the dispensation rates of ADHD medication and the types of ADHD medication dispensed per calendar year.
2. assess whether the dispensations of ADHD medication per calendar year were associated with dispensations of benzodiazepines, z-hypnotics, gabapentinoids, non-OAT opioids, as well as direct-acting antivirals (DAA) against hepatitis C infection, types of OAT opioids, the number of dispensed OAT opioids, gender, and age in the study period.
3. define the mean daily doses of dispensed ADHD medications, and the dispensation rates of benzodiazepines, z-hypnotics, gabapentinoids, and non-OAT opioids in 2017 among patients who were dispensed ADHD medication in the calendar year throughout the study period.

## Methods

### Data source

All data were register data and were drawn from the Norwegian Prescription Database (NorPD). From January 1, 2004, all pharmacies are obliged to submit data for all dispensed drugs electronically to NorPD underlying the Norwegian Institute of Public Health (www.norpd.no). The NorPD contains information on all drugs dispensed from pharmacies, except for drugs administrated at hospitals, nursing homes, and outpatient clinics [23]. The Anatomical Therapeutic Chemical (ATC) classification system was employed in accordance with the World Health Organization (WHO) standards per October 2018 [24].

### Study population

All patients above 18 years of age who received at least one dispensation of OAT opioids per calendar year, including methadone, levomethadone, buprenorphine, and buprenorphine-naloxone from January 1, 2015, to December 31, 2017, were included. In addition, some patients in palliative care use methadone tablets to achieve pain relief. These patients were excluded by identifying those who only were dispensed methadone tablets without any dispensations of other OAT opioids or methadone mixture in the period from January 1, 2004, to December 31, 2017.

### Analysis strategy and statistical analyses

#### Definitions of drugs, including ADHD medications and opioid agonist therapy opioids, the number of dispensations of OAT opioids, and diagnoses

Attention deficit hyperactivity disorder medications were defined as all CAS that had marketing authorizations in Norway in the period 2015 to 2017, including racemic amphetamine, dexamphetamine, methylphenidate, and lisdexamphetamine. In addition, we included the non-stimulant atomoxetine, which is also authorized and recommended in the treatment of ADHD according to guidelines and reviews [6–10, 18, 25]. For methylphenidate, the dispensations were classified by whether the formulation was ‘short- or intermediate-acting’ or ‘long-acting.’ Long-acting methylphenidate included depot formulations (Concerta®, Delmosart®, Equasym Depot®, or Methylphenidate Sandoz®), while short- or intermediate-acting methylphenidate included all other formulations (capsules or tablets). All included OAT opioids, ADHD medications, non-OAT opioids, benzodiazepines, z-hypnotics, gabapentinoids, including gabapentin and pregabalin, and DAAs were categorized according to their ATC codes (Additional file 1). The type of OAT opioid that patients were dispensed was defined as the last type of OAT opioid that was dispensed per calendar year.

The number of dispensed OAT opioids was defined as the number of dispensations of any OAT opioid per patient per calendar year. The number of dispensations was stratified according to four categories: 1–6, 7–12, 13–51, and ≥ 52 dispensations per calendar year. Age was defined according to the patient’s age in the calendar year and categorized into five groups: ≤ 25, 26–35, 36–45, 46–55, and ≥ 56 years.

All reimbursable and non-reimbursable ADHD medication dispensations were included. The prescribing physician needs to specify the medical condition that is treated by the particular drug, using codes from the 10th revision of International Classification of Diseases (ICD-10) or International Classification of Primary Care 2 (ICPC-2) to get public drug expenses reimbursed in Norway. The diagnostic codes of reimbursed drugs are recorded in the NorPD. Only two medical indications are approved for ADHD medication expense reimbursements in Norway: Hyperkinetic disorder/ADHD (ICD-10: F90 and ICPC-2: P81) or narcolepsy (ICD-10: G47 and ICPC-2: P81). For narcolepsy, only CAS are reimbursed. The information on diagnostic codes for non-reimbursable dispensations are not collected in the NorPD.

#### Analysis strategy according to the aims

One-year’s dispensation rates of ADHD medication during the study period were assessed by summing all patients who received at least one dispensation of an ADHD medication per the calendar year. Furthermore, patients were divided into two groups “all medical indications” and “ADHD” for the years in the study period. The group named “ADHD” only included patients who were dispensed ADHD medications with reimbursement codes for ADHD. The group named “all medical indications” included all patients who received dispensations of ADHD medications, either they were reimbursed or not. Less than five patients were dispensed CAS on the reimbursement code for narcolepsy in the study period.

The association with being dispensed ADHD medication, or not adjusted for age, gender, type of dispensed OAT opioids, the number of dispensed OAT opioids, being dispensed benzodiazepines, z-hypnotics, gabapentinoids, or non-OAT opioids were calculated per calendar year in the study period. Age and the number of dispensed OAT opioids were categorized according to the predefined categories or groups per year. All dispensed ADHD medication, and potentially addictive drugs were identified and categorized into four drug groups: “benzodiazepines or z-hypnotics,” “gabapentinoids,” “non-OAT opioids,” and “ADHD medication” per year. For each group, categorical variables were created by whether patients were dispensed one or more of the drugs in the drug groups or not. Dispensations of DAA were also included due to the frequent use of illicit stimulant drugs in the OAT population and the fact that DAA against hepatitis C infection has made treatment more applicable for these patients. Patients were defined to be dispensed treatment with DAA if they had at least one dispensation of DAA from 2011 and until the end of 2015, 2016, or 2017, respectively.

The mean daily dose of each ADHD medication and the dispensation rates of benzodiazepines or z-hypnotics, gabapentinoids, and non-OAT opioids in 2017, were calculated among patients with at least one dispensation of ADHD medication and OAT, respectively, for each calendar year in the study period. These patients were assumed to have achieved medical continuity in their ADHD treatment and follow up treatment according to national guidelines. The mean daily doses of ADHD medication were calculated by summarizing the total volume of defined daily doses (DDD) of each drug that was dispensed for each patient per year [26]. Further, the number of DDDs dispensed per patient was converted to milligrams according to WHO’s standards (Additional file 2). The mean daily dose for each ADHD medication was calculated by dividing the total dose (in milligram) of each drug per year by 365.25 days. The drug groups of each potentially addictive drugs were used to calculate dispensation rates. Each drug group was defined as categorical variables according to whether patients were dispensed at least one drug defined into the drug group or not during 2017.

#### Statistical analyses

Means, medians, percentiles, percentages, 95% confidence intervals (CI), odds ratios (OR), and *p*-values are presented when appropriate. Multivariable analyses for categorical variables were performed by binary logistic regression. Being dispensed ADHD medication, as well as OAT at least once, respectively, during a calendar year, were defined as a dependent variable in the logistic regression model. Independent variables were age, gender, ‘the number of dispensations of OAT opioids,’ ‘benzodiazepines or z-hypnotics,’ ‘gabapentinoids,’ ‘non-OAT opioids,’ and ‘DAA.’ All these variables were defined categorically. The level of statistical significance was defined as *p* < 0.05. The Chi-square test and paired sample t-test were used to estimate differences in dispensed mean daily doses of ADHD medication in 2015 compared to 2017 among patients with at least one dispensation of ADHD medication and OAT, respectively, during a calendar year throughout the study period. All patients were censored from the calendar year they died. SPSS version 24 was used for all analyses.

### Ethical considerations

The Regional Committee for Medical and Health Research Ethics, REC vest, Norway, has approved the use of registry data for this study (approval number 2018/939/REK Vest, June 19, 2018). No informed consent from included patients was necessary. The STROBE checklist was applied in the preparation of the study (Additional file 3).

## Results

### Baseline characteristics

A total of 9235 patients received at least one OAT opioid from pharmacies in Norway in the period 2015 to 2017. In 2017, 69% were male, and the mean age was 45 years (Table 1). A total of 376 participants died during the study period.

**Table 1:** Baseline characteristics

	2015		2016		2017	
Patients	7958		7804		7709	
Deaths	138		114		124	
Patients, excl. Deaths	7820		7690		7585	
	**OAT**	**OAT + AM**^b^	**OAT**	**OAT + AM**^b^	**OAT**	**OAT + AM**^b^
	No. (%)	No. (%)	No. (%)	No. (%)	No. (%)	No. (%)
Dispensed ADHD medication	–	274 (3.5)	–	312 (4.1)	–	349 (4.6)
Age						
≤ 25	171 (2.2)	5 (1.8)	135 (1.8)	7 (2.2)	120 (1.6)	9 (2.6)
26–35	1551 (19.8)	81 (29.6)	1403 (18.2)	90 (28.8)	1333 (17.6)	84 (24.1)
36–45	2605 (33.3)	107 (39.1)	2508 (32.6)	118 (37.8)	2392 (31.5)	134 (38.4)
46–55	2544 (32.5)	69 (25.2)	2540 (33.0)	79 (25.3)	2548 (33.6)	97 (27.8)
≥ 56	949 (12.1)	12 (4.4)	1104 (14.4)	18 (5.8)	1192 (15.7)	25 (7.2)
*Mean (SD)*	*43.9 (9.7)*	*41.0 (8.5)*	*44.5 (9.8)*	*40.8 (8.7)*	*45.0 (9.9)*	*41.8 (9.0)*
Gender						
Male	5430 (69.4)	193 (70.4)	5354 (69.6)	221 (70.8)	5245 (69.1)	254 (72.8)
Female	2390 (30.6)	81 (29.6)	2336 (30.4)	91 (29.2)	2340 (30.9)	92 (26.4)
OAT opioids^a^						
Methadone (included levomethadone)	3216 (41.1)	72 (26.3)	3066 (39.9)	74 (23.7)	2981 (39.3)	92 (26.4)
Buprenorphine (included combinations)	4604 (58.9)	202 (73.7)	4624 (60.1)	238 (76.3)	4604 (60.7)	257 (73.6)

*ADHD* Attention deficit hyperactivity disorder, *AM* ADHD medication (atomoxetine, racemic amphetamine, dexamphetamine, lisdexamphetamine, and methylphenidate), *NorPD* Norwegian Prescription Database, *SD* standard deviation, and *No* Number of patients

^a^ The last dispensed OAT opioid in the calendar year

^b^ On all medical indications

The table displays the baseline characteristics of patients who were dispensed at least one OAT opioid per year in the period from 2015 to 2017

### One-year prevalence and the types of dispensed ADHD medications

The proportions of OAT patients who received at least one dispensation of an ADHD medication increased from 3.5% in 2015 to 4.6% in 2017. A vast majority of them, i.e., 74% received buprenorphine or buprenorphine-naloxone, whereas the remaining 26% were dispensed methadone or levomethadone. In 2017, the most dispensed CAS was short- and intermediate-acting methylphenidate (55%), followed by lisdexamphetamine (24%), dexamphetamine (17%), long-acting methylphenidate (9%), and racemic amphetamine (2%) (Table 2ab). The non-stimulant atomoxetine was dispensed in 6% of these patients. These findings were substantially similar to the results in 2015 and 2016.

**Table 2:** The proportion of patients on OAT were dispensed ADHD medication categorized on medical diagnoses and types of CAS

a)						
**Year**	**2015**		**2016**		**2017**	
	**All indications**					
Number of patients	274		312		349	
**ADHD medication**	*No.*	*%*^a^	*No.*	*%*^a^	*No.*	*%*^a^
Methylphenidate	194	71	217	70	207	59
- short- and intermediate-acting^b^	*182*	*66*	*206*	*66*	*193*	*55*
- long-acting^c^	*38*	*14*	*30*	*10*	*33*	*9*
Dexamphetamine	63	23	64	21	60	17
Atomoxetine	23	8	26	8	21	6
Lisdexamphetamine	14	5	47	15	84	24
Racemic amphetamine	< 5	0	< 5	1	8	2
b)						
**Year**	**2015**		**2016**		**2017**	
	**ADHD**					
Number of patients	223		270		312	
**ADHD medication**	*No.*	*%*^a^	*No.*	*%*^a^	*No.*	*%*^a^
Methylphenidate	171	76	198	72	194	62
- short- and intermediate-acting^b^	*163*	*73*	*190*	*70*	*182*	*58*
- long-acting^c^	*32*	*14*	*26*	*10*	*29*	*9*
Dexamphetamine	45	20	55	20	53	17
Atomoxetine	12	5	19	7	16	5
Lisdexamphetamine	12	5	39	14	72	23
Racemic amphetamine	< 5	< 5	< 5	< 5	7	2

*ADHD* Attention deficit hyperactivity disorder, *ADHD* medication = atomoxetine, racemic amphetamine, dexamphetamine, lisdexamphetamine, and methylphenidate, *ICD-10* 10th Revision of International Classification of Diseases, *ICPC2* International Classification of Primary Care 2, and *OAT* opioid agonist therapy

^a^ Per cent of patients who received OAT and CAS, ^b^ Include all tablets and capsules with short- and intermediate-acting methylphenidate, ^c^ Include depot formulations of methylphenidate (Concerta®, Delmosart®, Equasym Depot®, or Methylphenidate Sandoz®)

The tables display patients on OAT who were dispensed an ADHD medication in the period 2015 to 2017 categorized on a) all medical indications, and b) ADHD (ICD-10 code: F90 or ICPC2 code: P81)

### Dispensations of potentially addictive drugs to patients receiving OAT opioids and ADHD medication concomitantly

In the period from 2015 to 2017, being dispensed ADHD medications were associated with being dispensed buprenorphine rather than methadone as OAT opioid (2017: adjusted odds ratio (aOR): 1.6, 95% confidence interval (CI): 1.3–2.1) (Table 3). Further, in 2017, being dispensed ADHD medications were associated with being dispensed a non-OAT opioid (aOR 1.5, 95% CI: 1.1–1.9) and a DAA against hepatitis C infection (aOR 1.6, 95% CI: 1.2–2.2). The odds ratio (OR) of being dispensed DAA increased steadily during the study period. Being dispensed ADHD medications were not statistically associated with being dispensed gabapentinoids, benzodiazepines, or z-hypnotics per year in the study period.

**Table 3:** Logistic regression analyses of variables associated with dispensed ADHD medication and OAT

	2015		2016		2017	
**Dispensed ADHD medication**	*N* = 274		*N* = 312		*N* = 349	
**Not dispensed ADHD medication**	*N* = 7546		*N* = 7378		*N* = 7236	
	**Crude OR**	**Adjusted OR (95% CI)**	**Crude OR**	**Adjusted OR (95% CI)**	**Crude OR**	**Adjusted OR (95% CI)**
Age						
- ≤ 25	1.0 (ref.)	1.0 (ref.)	1.0 (ref.)	1.0 (ref.)	1.0 (ref.)	1.0 (ref.)
- 26–35	1.8	1.8 (0.7–4.5)	1.3	1.3 (0.6–2.9)	0.8	0.8 (0.4–1.7)
- 36–45	1.4	1.5 (0.6–2.7)	0.9	1.0 (0.5–2.2)	0.7	0.7 (0.4–1.5)
- 46–55	0.9	1.0 (0.4–2.6)	0.6	0.7 (0.3–1.6)	0.5	0.5 (0.2–1.0)
- ≥ 56	0.4	0.5 (0.2–1.4)	0.3	0.4 (0.2–0.9)	0.3	*0.3 (0.1–0.6)*
Gender						
- Female	1.0 (ref.)	1.0 (ref.)	1.0 (ref.)	1.0 (ref.)	1.0 (ref.)	1.0 (ref.)
- Male	1.1	1.0 (0.8–1.4)	1.1	1.0 (0.8–1.3)	1.2	1.2 (1.0–1.6)
The number of dispensations of OAT opioids per calendar year						
- ≥ 52	1.0 (ref.)	1.0 (ref.)	1.00 (ref.)	1.0 (ref.)	1.0 (ref.)	1.00 (ref.)
- 13–51	0.9	1.0 (0.7–1.4)	0.7	0.8 (0.6–1.2)	1.1	1.1 (0.8–1.7)
- 7–12	0.7	0.8 (0.5–1.3)	0.8	0.9 (0.6–1.3)	1.3	1.3 (0.9–2.0)
- 1–6	0.7	0.7 (0.4–1.2)	0.9	1.0 (0.7–1.6)	1.0	1.0 (0.6–1.7)
OAT opioids^a^						
- Methadone (incl. Levomethadone)	1.0 (ref.)	1.00 (ref.)	1.00 (ref.)	1.0 (ref.)	1.0 (ref.)	1.0 (ref.)
- Buprenorphine (incl. combinations)	*2.0*	*1.7 (1.3–2.3)*	*2.2*	*1.9 (1.4–2.5)*	*1.9*	*1.6 (1.3–2.1)*
Dispensed opioids (excl. OAT opioids)	1.0	1.1 (0.8–1.5)	1.0	1.1 (0.8–1.5)	*1.4*	*1.5 (1.1–1.9)*
Dispensed gabapentinoids	0.8	0.9 (0.5–1.4)	1.2	1.2 (0.8–1.7)	1.1	1.0 (0.7–1.4)
Dispensed benzodiazepines and/or z-hypnotics	0.8	1.0 (0.7–1.2)	0.8	0.9 (0.7–1.2)	1.0	1.0 (0.8–1.3)
Dispensed DAA	0.8	1.0 (0.5–2.1)	1.1	1.4 (0.9–2.3)	*1.4*	*1.6 (1.2–2.2)*

*ADHD* Attention deficit hyperactivity disorder, *ADHD medication* Atomoxetine, racemic amphetamine, dexamphetamine, lisdexamphetamine, and methylphenidate, *CI* confidence interval, *DAA* direct-acting antivirals against hepatitis C infection, and *OAT* opioid agonist therapy

^a^ The last OAT opioid was dispensed during a calendar year recorded in the Norwegian Prescription Database

The table displays odds ratios for each independent variable among patients who were dispensed ADHD *medication* (dependent variable) and OAT. For example, the adjusted odds of being dispensed opioids in 2017 was 1.5 among patients who were dispensed ADHD *medication*. Each independent variable is stated as crude (unadjusted) and adjusted for each calendar year. Italics display significant values

### Mean daily doses of dispensed ADHD medications and dispensation rates of other potentially addictive drugs

**Fig. 1:**
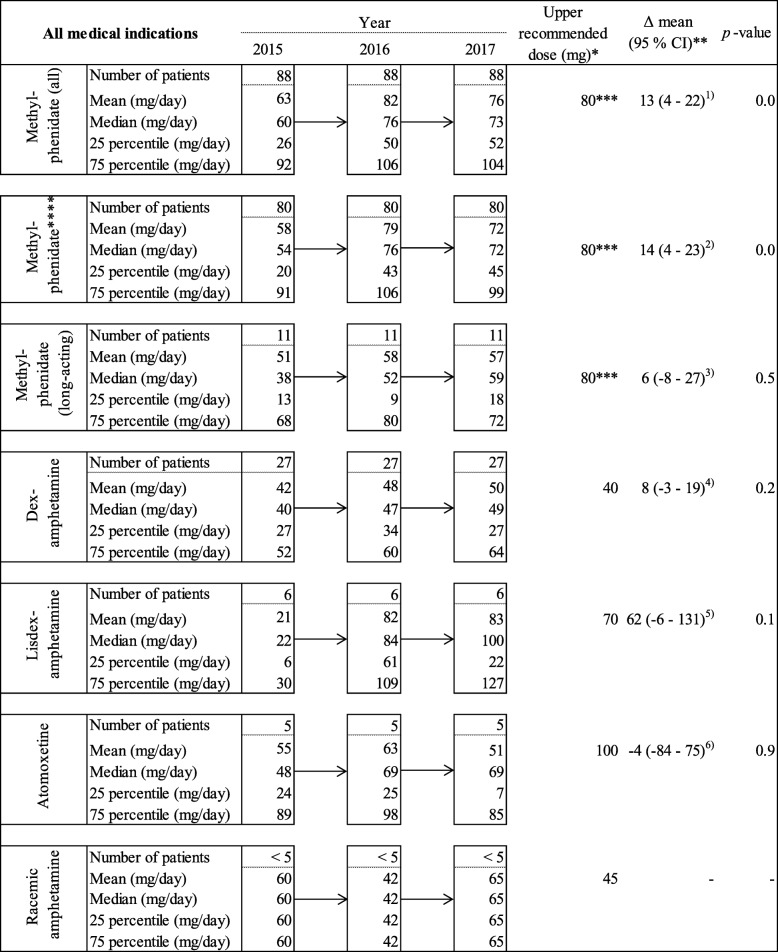
Doses of dispensed ADHD medication among patients who received OAT opioids from 2015 to 2017. Legends: ADHD = Attention deficit hyperactivity disorder, ADHD medication = atomoxetine, racemic amphetamine, dexamphetamine, lisdexamphetamine, and methylphenidate, CI = confidence interval, and Df = degrees of freedom. ^1)^ Paired-samples t-test, df = 87, ^2)^ Paired-samples t-test, df = 79, ^3)^ Paired-samples t-test, df = 10, ^4)^ Paired-samples t-test, df = 26, ^5)^ Paired-samples t-test, df = 5, and ^6)^ Paired-samples t-test, df = 4. * = Upper recommended doses according to The European Medicines Agency (EMA) per July 2019, ** = Calculation of the differences in mean daily doses between 2015 and 2017, *** = Upper recommended daily dose of short- and intermediate-acting methylphenidate according to the EMA, and **** = Include short- and intermediate-acting methylphenidate (tablets or capsules), not depot formulations. The figure displays the mean daily doses of each dispensed ADHD medication among patients who were dispensed at least one dispensation ADHD medication and OAT opioid, respectively, each calendar year in the study period from 2015 to 2017

**Fig. 2:**
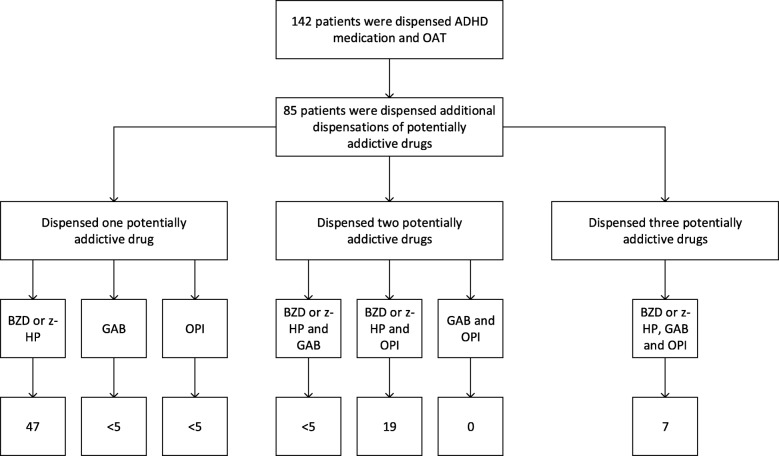
Patients on ADHD medication who were dispensed other potentially addictive drugs in 2017. Legends: ADHD = Attention deficit hyperactivity disorder, ADHD medication = atomoxetine, racemic amphetamine, dexamphetamine, lisdexamphetamine, and methylphenidate, BZD = benzodiazepines, GAB = gabapentinoids, OAT = Opioid agonist therapy, OPI = non-OAT opioids, and z-HP = z-hypnotics. The figure displays dispensations of BZD, GAB, OPI, and z-HYP in 2017 among patients who were dispensed at least one dispensation of ADHD medication and OAT opioids each calendar year in the period from 2015 to 2017. Eighty-five patients were dispensed BZD or z-HP, GAB, or OPI in this population

We identified 142 patients who received at least one dispensation of ADHD medication per calendar year throughout the study period. We found a substantial increase in the dispensed mean daily doses of methylphenidate from 63 mg in 2015 to 76 mg in 2017 (*p* = 0.01) (Fig. 1). The mean doses of other dispensed ADHD medications were not statistically significantly different in 2017 compared to 2015. However, the mean daily dose of lisdexamphetamine increased from 21 mg in 2015 to 83 mg in 2017. The mean doses of amphetamine, dexamphetamine, methylphenidate, and lisdexamphetamine were near the highest recommended doses for each drug, according to the European Medicines Agency (EMA) [27]. Furthermore, 85 of the 142 patients (60%) who were dispensed an ADHD medication per year throughout the study period also received at least one dispensation of z-hypnotics or benzodiazepines, gabapentinoids, or non-OAT opioids in 2017 (Fig. 2). The most frequent combination of dispensed drugs was ‘OAT opioid, ADHD medication, and benzodiazepines, or z-hypnotics.’ Seven patients received ‘OAT opioid, ADHD medication, benzodiazepines, or z-hypnotics, gabapentinoids, and non-OAT opioids.’

## Discussion

In the period 2015 to 2017, the proportion of patients receiving ADHD medication in the OAT population increased from 3.5 to 4.6%. Short- and intermediate-acting methylphenidate and lisdexamphetamine were the most frequently dispensed CAS. Dispensation of buprenorphine rather than methadone as an OAT opioid was associated with being dispensed ADHD medication. In 2017, being dispensed non-OAT opioids and DAA against hepatitis C infection were associated with being dispensed ADHD medication. For four out of five ADHD medication, the mean doses were near the highest recommended doses. Furthermore, the dose of methylphenidate increased significantly throughout the study period. Eighty-five of 142 patients who were dispensed ADHD medication each year throughout the study period were also dispensed benzodiazepines, z-hypnotics, gabapentinoids, or non-OAT opioids in 2017.

Short- and intermediate-acting methylphenidate is the most dispensed CAS throughout the study period. These formulations, particularly the short-acting formulation, are associated with euphoria and addiction compared to long-acting formulation [18, 28]. However, the short- and intermediate-acting methylphenidate might be preferable in situations where more focus and concentration is needed for shorter periods. In Norway, the reimbursement for methylphenidate for adults is pre-approved for intermediate-acting formulations as opposed to long-acting formulations [29], which may explain that few patients were dispensed long-acting formulations. A study evaluating the dispensations of ADHD medications in the general population among the Nordic countries showed that Denmark and Norway, in contrast to Finland, Iceland, and Sweden, were substantially dispensed intermediate-acting rather than long-acting methylphenidate in the treatment of ADHD [30]. The Norwegian guidelines for treating ADHD do not mention the formulation of methylphenidate to patients on OAT in their recommendations [7]. However, a European consensus report recommends long-acting formulations of CAS to prevent misuse among patients with drug use disorders and ADHD [18].

The proportion of patients who received ADHD medication increased in the inclusion period. Nevertheless, the dispensation rates were still in the lower range of what was expected. It is estimated that as much as a third of patients with drug addictions in Norway have comorbid ADHD [16], and the proportion of those with opioid use disorder is supposed to be 11–33% [3–5]. Assuming that 40–50% of patients with ADHD were dispensed ADHD medication in the general population [18], one would expect that about 4–16% of those with opioid use disorder receive ADHD medication. Our findings showed that only 4–5% of the patients on OAT also were dispensed ADHD medication during the study period. This might have several explanations. A consensus report evaluating screening, diagnosis, and treatment of patients with drug use disorders and ADHD, recommends the use of CAS when potentially therapeutic pros and cons are considered in advance [18]. In addition, the Norwegian guidelines for ADHD discourage dispensations of ADHD medication to patients on OAT who used other potentially addictive drugs concomitantly [7]. Furthermore, low dispensation rates of CAS may also be explained by medical illnesses or psychosocial conditions, and active illicit drug use, which may disturb the diagnostic assessment of ADHD and delay pharmacological treatment.

Retention to treatment is generally challenging in the treatment of drug addictions, particularly among patients with comorbid ADHD. Inadequate knowledge of pharmacological properties of different ADHD medications may explain a low coverage. For example, unlike CAS, the non-stimulant atomoxetine may need several weeks to give optimal clinical response [18]. Late-onset of the effect of atomoxetine or careful dose-escalation of methylphenidates and amphetamines may conflict with patient’s expectations on a quick reduction of ADHD symptoms. In addition, removing factors leading to discontinuation of CAS treatment may play an essential role in preventing relapse to illicit stimulant drug use and sustained stimulant injections, as well as improving the quality of life by keeping complications such as hepatitis C infection low [31]. Integrating ADHD treatment in OAT, or vice versa, maybe a way to facilitate the diagnostics and treatment and improve follow-up approaches among marginalized patients on OAT with comorbid ADHD [32].

In this study, the mean doses of ADHD medications were in the highest range of usual recommended doses. The benefits of high-dose ADHD medication on the treatment of ADHD in the OAT population are not clear. Two placebo-controlled randomized trials, including patients with ADHD and addictions to amphetamines or cocaine, have found a decrease of ADHD symptoms by using doses up to 180 mg methylphenidate [19] and 80 mg racemic amphetamine daily compared to placebo [20]. The former study [19] also found that high-dose of methylphenidate reduced relapse to illicit stimulant use and contributed to higher retention in treatment. Previous research has also confirmed similar findings [33]. The latter study [20], evaluating racemic amphetamine to placebo, showed that doses of 60 mg and 80 mg racemic amphetamine per day, respectively, inhibited cocaine-related craving. Although, a dose of 80 mg racemic amphetamine did not seem to reduce ADHD symptoms more than a dose of 60 mg per day. Overall, one can assume that using higher doses of methylphenidate or racemic amphetamine may improve the effect of these medications on ADHD by keeping patients in treatment, reducing the craving for illicit stimulant drugs, as well as by alleviating ADHD symptoms.

The proportion of patients who were dispensed lisdexamphetamine increased significantly from 2015 to 2017. In addition, the mean dose rose markedly in the same period, although it was not statistically significant. A meta-analysis evaluating the efficacy, acceptability, and tolerability of ADHD medication among patients with ADHD without drug addiction favored amphetamines as the first drug group of choice in the short-term treatment of ADHD in adults [25]. By comparing methylphenidate and amphetamines, the latter was more efficacious and showed higher acceptability (i.e., fewer patients leaving the study). National Institute for Health and Care Excellence (NICE) guidelines [9] and a consensus report [18] evaluating patients with drug addictions and ADHD recommend methylphenidate or lisdexamphetamine as the first drugs of choice in the treatment of ADHD in adults. A risk assessment of the potential of misuse of lisdexamphetamine and methylphenidate has been completed by the WHO, which pointed towards that methylphenidate and lisdexamphetamine still have low harmful profiles compared to other stimulants such as racemic amphetamine and methamphetamine in treatment of ADHD [34]. The use of ADHD medication in the Norwegian OAT population was in line with these recommendations. In addition, it is essential to mention that the lisdexamphetamine named Aduvanz® was granted the Norwegian marketing authorization in September 2017, and the upcoming facilitation in the pre-approved reimbursement of lisdexamphetamine was introduced in October 2018 [35]. These changes may also explain some of the increasing dispensation rates found in this study.

Eighty-five of 142 patients who were dispensed ADHD medication and OAT opioids concomitantly received either benzodiazepines, z-hypnotics, gabapentinoids, or non-OAT opioids at least as frequent as in the remaining OAT population not were dispensed ADHD medications in 2017. Our findings confirm previous studies on the OAT population, showing that about half of patients on OAT were dispensed other potentially addictive drugs [11, 36]. The fact that a substantial proportion of patients were dispensed CAS concomitant with dispensations of other potentially addictive drugs may point towards the need to improve the prescribing practice of addictive drugs in this comorbid population in order to keep the risk of adverse interactions low [11, 12, 37]. On the other hand, the prevalence of psychiatric and somatic comorbidities in OAT is high [38–40], and it may predict the high dispensation rates of potentially addictive drugs when these comorbidities are treated. In some cases, prescribing potentially addictive drugs may be used to keep the patients completely abstinent from illicit potentially addictive drugs if health professionals follow up strictly, and the prescribing practices are considered proper [14, 15].

## Strengths and limitations

The use of national registry data has some clear strengths, by capturing whole cohorts of the studied populations. Pharmacy records are considered more valid than both medical records and data collected from questionnaires and interviews. Because practically all dispensed drugs are registered in the NorPD database, completeness, and precision of all received information is high, and the potential for information biases is low.

This study also had some limitations. First, because non-reimbursed dispensations of ADHD medication were not received through the Norwegian Health Economics Administration (HELFO), the medical indications for these dispensations are not available for the researchers through NorPD. Further, gabapentinoids, benzodiazepines, z-hypnotics, and non-OAT opioids have different medical indications, and the indications have not been evaluated in this study. Second, the number of dispensed OAT opioids may be incompletely registered by the pharmacies. The self-reporting survey of OAT in Norway in 2017 showed that the mean frequency of dispensations of OAT opioids was four times a week [22]. This finding may indicate that the number of dispensations is underestimated. Third, the NorPD only receives information about dispensed drugs, and we cannot know whether the drugs have been consumed. All addictive drugs are coveted for illegal consumption, and the drugs may be re-distributed. Illicit use is common in this population and cannot be covered using register data. Fourth, slightly less than 10% of OAT opioids are dispensed in addiction specialist outpatient clinics, and those are not necessarily registered in the NorPD. Some of these outpatient clinics order OAT opioids directly from pharmacies without linking to a personal identification number. These patients were missed in this study, and those could have higher dispensation rates of addictive drugs than patients included in this study [22].

## Conclusion

Co-prescribing of CAS and atomoxetine was low in the OAT population in Norway, relative to the expected prevalence of ADHD in this patient group. Considering that up to a third of the OAT population is estimated to have ADHD, only 3.5 to 4.6% of patients received both ADHD medication and OAT opioids in Norway in the period from 2015 to 2017. Furthermore, 85 of 142 OAT patients who were dispensed ADHD medication each year throughout the study period were dispensed at least one dispensation of other potentially addictive drugs concomitantly in 2017. Generally, the polydrug use, including CAS, OAT, and other potentially addictive drugs, may lead to adverse side effects; however, a treatment combining several potentially addictive drugs in OAT patients using CAS has only been scarcely studied. Randomized-controlled trials evaluating ADHD medication in different doses are needed to improve the treatment of ADHD in the OAT population.

## Supplementary information

**Additional file 1.** ATC codes for included drugs, or drug groups. ADHD = Attention deficit hyperactivity disorder, ATC = Anatomical Therapeutic Chemical (ATC) classification system, and OAT = opioid agonist therapy. An overview of drugs and drug groups included in this study.

**Additional file 2.** Converting table of DDD to milligrams according to WHO’s standards. ATC = The Anatomical Therapeutic Chemical (ATC) classification system, ADHD = Attention deficit hyperactivity disorder, DDD = defined daily doses, mg = milligram, WHO = World Health Organization.

**Additional file 3.** STROBE Statement. Checklist of items that should be included in reports of cohort studies

## Data Availability

Except supplemental tables with some additional data, no additional data are available due to data protection requirements.

## Abbreviations

ADHD: Attention deficit hyperactivity disorder
AOR: Adjusted odds ratio
ATC: Anatomical Therapeutic Chemical
CAS: Centrally acting stimulants
CI: Confidence interval
DAA: Direct-acting antivirals
DDD: Defined Daily Doses
EMA: The European Medicines Agency
HELFO: The Norwegian Health Economics Administration
ICD-10: 10th revision of International Classification of Diseases
ICPC 2: International Classification of Primary Care 2
NICE: National Institute for Health and Care Excellence
NorPD: The Norwegian Prescription Database
OAT: Opioid agonist therapy
OR: Odds ratio
WHO: World Health Organization
